# The medium-chain fatty acid decanoic acid reduces oxidative stress levels in neuroblastoma cells

**DOI:** 10.1038/s41598-021-85523-9

**Published:** 2021-03-17

**Authors:** Janine Mett, Uli Müller

**Affiliations:** Biosciences Zoology/Physiology-Neurobiology, ZHMB (Center of Human and Molecular Biology) Faculty NT-Natural Science and Technology, Saarland University, 66123 Saarbrücken, Germany

**Keywords:** Cell biology, Neuroscience

## Abstract

Enhanced oxidative stress is a contributing factor in the pathogenesis of several neurodegenerative disorders such as Alzheimer´s disease. Beneficial effects have been demonstrated for medium-chain fatty acids (MCFAs) nutritionally administered as medium-chain triglycerides (MCTs) or coconut oil (CO). The observed effects on cognitive impairment are generally attributed to the hepatic metabolism of MCFAs, where resulting ketone bodies serve as an alternate energy source to compensate for the impaired glucose utilisation in the human brain. Here we show that the saturated MCFA decanoic acid (10:0) reduces the oxidative stress level in two different neuroblastoma cell lines. Phosphatidylcholine (PC) containing decanoic acid (10:0) (PC10:0/10:0) reduced the cellular H_2_O_2_ release in comparison to solvent, L-α-Glycerophosphorylcholine and PC containing the long-chain fatty acid (LCFA) arachidic acid (20:0). This effect seems to be at least partially based on an upregulation of catalase activity, independent of alterations in catalase gene expression. Further, PC10:0/10:0 decreased the intracellular oxidative stress level and attenuated the H_2_O_2_-induced cell death. It did not affect the level of the ketone body β-hydroxybutyrate (βHB). These results indicate that decanoic acid (10:0) and possibly MCFAs in general directly reduce oxidative stress levels independent of ketone levels and thus may promote neuronal health.

## Introduction

Oxidative stress is defined as ‘an imbalance in pro-oxidants and antioxidants with associated disruption of redox circuitry and macromolecular damage’^1^. It results in elevated levels of reactive oxygen and nitrogen species (ROS, RNS) including O_2_^•−^ (superoxide radical anion), OH^•^ (hydroxyl radical), H_2_O_2_ (hydrogen peroxide), ^•^NO (nitric oxide) and ONOO^−^ (peroxynitrite)^2^. ROS are physiologically derived from the mitochondrial electron transport chain and the activity of NADPH oxidases (NOXs) transferring electrons from NADPH across cellular membranes to molecular oxygen. The main ROS generated by NOX isoenzymes is O_2_^•−^, NOX4 predominantly releases H_2_O_2_. O_2_^•−^ is rapidly converted to H_2_O_2_ by superoxide dismutases (SODs) or to OH^•^. Cellular proportion of ROS generation and detoxification by antioxidant enzymes such as SODs, glutathione peroxidase (GPx) and catalase determines the ROS level^2–5^. Although ROS and in particular H_2_O_2_ might function in various signal transduction pathways, excessive levels of these free radicals are hazardous^5^. Oxidative damage of proteins, lipids and nucleic acids may eventually result in cellular degeneration, functional decline and cell death. The brain is particularly susceptible to oxidative injuries owed to its limited antioxidant capacity, its high energy demand and its high content of polyunsaturated fatty acids (PUFAs). Increased oxidative stress has been identified as a contributing factor in the pathogenesis of several neurodegenerative disorders, for example Alzheimer´s disease. Thus, the reduction of cerebral ROS level might help to slow down the progression of these diseases^2,6,7^.

Medium-chain fatty acids (MCFAs) are saturated fatty acids consisting of 6 to 12 carbon atoms. Natural dietary sources of these fatty acids are coconut oil (CO) and palm kernel oil with 62–70% MCFAs of the saturated fatty acid portion (> 90%). In contrast to other fatty acids MCFAs are easily absorbed and metabolized within liver mitochondria to produce the ketone bodies 3-β-hydroxybutyrate (βHB), acetoacetic acid (AcAc) and acetone (Ac)^8–11^. As they can be converted to acetyl-CoA, a key substrate in the citric acid cycle to provide ATP, ketone bodies can improve the cerebral energy metabolism during periods with limited glucose availability or uptake^10,12^. Nutritional studies in patients with minor to moderate cognitive impairment suggest the daily consumption of MCFAs, in the form of CO or medium-chain triglycerides (MCTs) to have beneficial effects. The observed effects are generally attributed to the elevation of circulating ketone bodies compensating for the cerebral glucose hypometabolism in patients suffering from Alzheimer´s disease^8,9,13–23^. In this context Wang et al*.* showed MCFAs and especially 10-carbon length MCTs to have neuroprotective and cognition-enhancing properties in aged Wistar rats, independent of brain ketone levels^24^.

In recent in vitro studies CO as well as pure MCFAs were found to protect primary cortical neurons from amyloid-β (Aβ)-induced toxicity. CO was demonstrated to diminish markers of oxidative stress in these cells^25,26^. Similarly, ketone bodies metabolically derived from CO and MCFAs have been reported to prevent oxidative injury in primary neurons and in SH-SY5Y cells^27,28^. Although advances in understanding the mechanisms of MCFA-action have more recently shifted attention away from ketone bodies to the direct role of fatty acids^29^, the effect of MCFAs on the oxidative stress level of neuronal cells is largely unknown. This prompted us to investigate the impact of the MCFA decanoic acid (10:0) on ROS levels in two different neuroblastoma cell lines, in which ketogenesis is assumed to be absent^30^.

## Results

### The MCFA decanoic acid (10:0) reduces H_2_O_2_ release from neuroblastoma cells

In order to analyze whether the MCFA decanoic acid (10:0) influences the oxidative stress level in neuronal cells, we treated neuroblastoma cells with 10 µM of the most abundant phospholipid in mammalian cellular membranes^31^, phosphatidylcholine (PC) containing decanoic acid (10:0) in the sn-1 and sn-2 position (1,2-didecanoyl-sn-glycero-3-phosphocholine, PC10:0/10:0). Control cells were treated with the solvent ethanol (EtOH) (set as 100%). As an additional reference we used PC containing the long-chain fatty acid (LCFA) arachidic acid (20:0) (1,2-Di-arachidoyl-sn-glycero-3-phosphocholine, PC20:0/20:0, final concentration 10 µM). This setup allowed us to determine the sole effect of the fatty acid chain length.

10 µM final concentration of the lipids reflects the plasma 10:0-concentration in rats fed a MCT-enriched diet (5% MCT containing decanoic acid (10:0)) for 8 weeks^24^. In order to lower the lipid content of the medium and to inhibit cell proliferation, the concentration of fetal calf serum (FCS) in the cell culture medium was reduced to 0.1% during the phospholipid treatment.

After 18 h (hours) treatment, for one the level of H_2_O_2_ accumulating in the medium during the treatment was measured by adding Amplex Red (10-acetyl-3,7-dihydroxyphenoxazine) and horseradish peroxidase (HRP) to the removed cell culture supernatant. In the presence of peroxidase, the Amplex Red reagent reacts stoichiometrically with H_2_O_2_ in a 1:1 ratio to produce the red-fluorescent oxidation product resorufin^32^. The outcomes of this assay reflect the extracellular steady state level of H_2_O_2_, which is dependent on the generation, flux and removal rate of H_2_O_2_ within the 18 h of treatment. Secondly the H_2_O_2_-release-activity of the cells was directly measured after supernatant removal and replacement with supplement-free medium containing the reactants Amplex Red and HRP.

**Figure 1:**
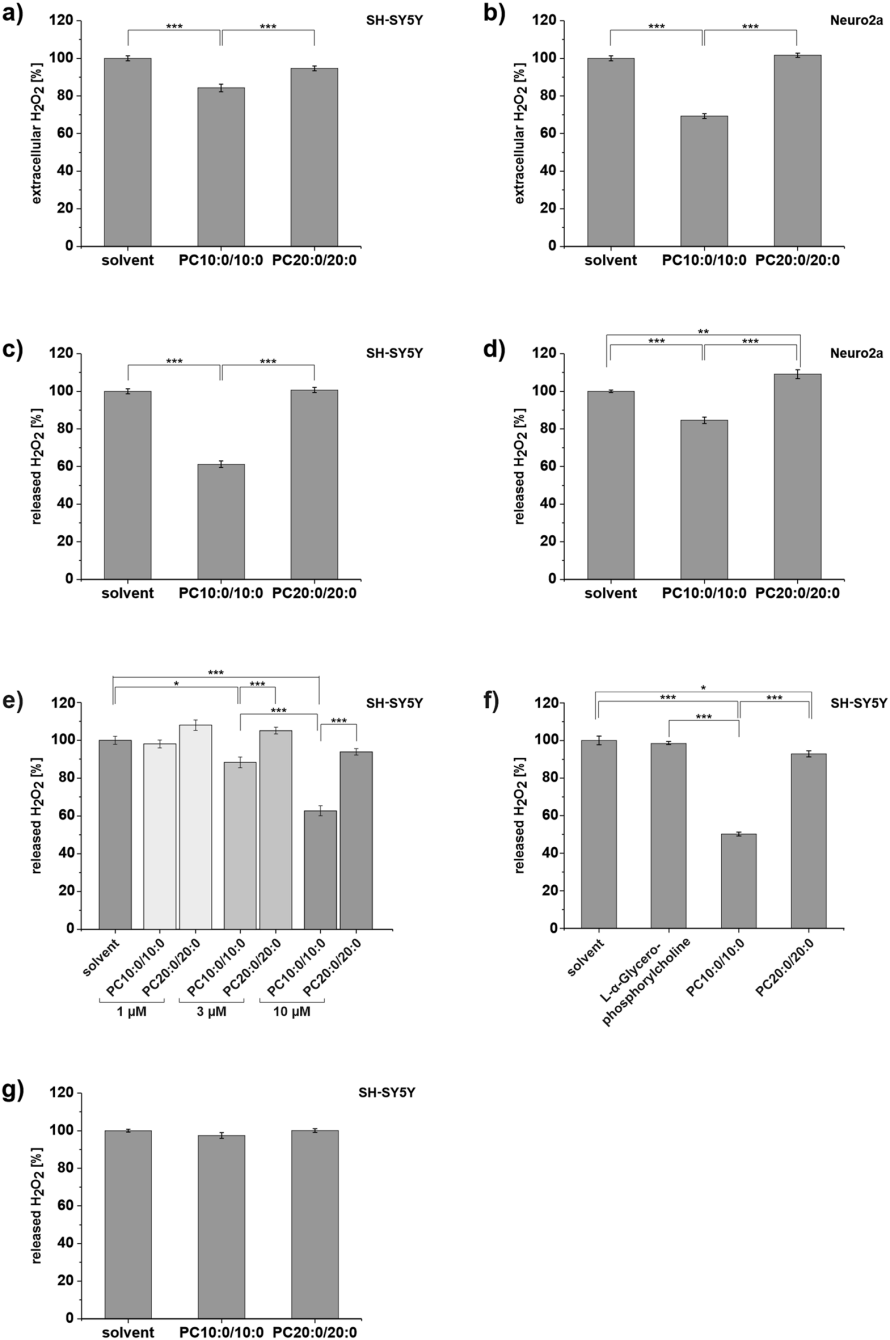
Impact of PC10:0/10:0, PC20:0/20:0 or solvent on the extracellular H_2_O_2_ level and H_2_O_2_ release. Cells were incubated with the solvent EtOH (0.2%) (set as 100%), PC10:0/10:0, PC20:0/20:0 (10 µM except for e) for 18 h (except for g) before H_2_O_2_ accumulating in the medium during the treatment and H_2_O_2_ release was measured by using Amplex Red (5 µM) and HRP (0.01 U/ml). 10 µM L-α-Glycerophosphorylcholine was used as an additional reference (f, 10 µM). H_2_O_2_ level in the conditioned medium of (**a)** SH-SY5Y cells (n = 12) and (**b)** Neuro2a cells (n = 8). H_2_O_2_ released by (**c)** SH-SY5Y cells (n = 28), (**d)** Neuro2a cells (n = 8), (**e)** SH-SY5Y cells treated with the solvent EtOH (0.2%) or different concentrations (1, 3 and 10 µM) of PC10:0/10:0 or PC20:0/20:0 (n ≥ 6), (**f)** SH-SY5Y cells additionally treated with 10 µM l-α-Glycerophosphorylcholine as reference (n = 6) and (**g)** SH-SY5Y cells after short-term treatment for 30 min (n = 14). Error bars represent SEM. Asterisks show the statistical significance calculated by one-way ANOVA followed by post hoc testing using Tukey's test (* p ≤ 0.05, ** p ≤ 0.01 and *** p ≤ 0.001). Figure was created using Origin Pro 2020b and CorelDRAW Graphics Suite 2020.

Our results revealed a significant reduction of the H_2_O_2_ level in the conditioned medium for human SH-SY5Y cells treated with PC10:0/10:0 in comparison to solvent (p = 1.47 × 10^–7^) and to PC20:0/20:0 (p = 1.61 × 10^–4^). PC20:0/20:0 had no significant effect on the extracellular H_2_O_2_ content compared to solvent (p = 0.065) (Fig. 1a). In order to verify this effect by another cell model, the experiment was replicated utilizing the murine neuroblastoma cell line Neuro2a. The effects were even more pronounced in the murine cells (PC10:0/10:0 vs. solvent: p < 1 × 10^–14^; PC10:0/10:0 vs. PC20:0/20:0: p < 1 × 10^–14^) (Fig. 1b). To rule out any interference between the added agents and the measured fluorescence or the HRP-catalyzed reaction of Amplex Red with H_2_O_2_, the experiment was repeated in a H_2_O_2_-supplemented, cell-free medium. There was no indication for fluorescence quenching or an inhibitory effect of PC10:0/10:0 on the reaction itself in the cell-free system. The detected fluorescence signal slightly increased in presence of this phospholipid compared to solvent (p = 0.020) and PC20:0/20:0 (p = 0.004) (Supplementary Fig. S1). Consequently, the observed effects of PC10:0/10:0 on the extracellular H_2_O_2_ level (Fig. 1a,b) are exclusively due to the presence of cells.

The H_2_O_2_-release measurement in the replacement medium showed that pretreatment of SH-SY5Y cells with PC10:0/10:0 significantly suppressed the natural release of H_2_O_2_ in comparison to both solvent (p < 1 × 10^–14^) and PC20:0/20:0 (p < 1 × 10^–14^). Again, H_2_O_2_ release did not differ between cells pretreated with solvent or PC20:0/20:0 (p = 0.943) (Fig. 1c). As shown in Fig. 1d, the effect of PC10:0/10:0 on H_2_O_2_ release could be verified in the murine Neuro2a cells (PC10:0/10:0 vs. solvent: p = 9.55 × 10^–6^; PC10:0/10:0 vs. PC20:0/20:0: p < 1 × 10^–14^). There was also a slight, but significant increase of H_2_O_2_ release in Neuro2a cells preincubated with PC20:0/20:0 compared to solvent (p = 0.004). Accordingly, PC10:0/10:0 reduces the H_2_O_2_ release of both SH-SY5Y and Neuro2a cells resulting in a declined steady state level of H_2_O_2_ in the conditioned cell culture supernatant (Fig. 1a,b).

In order to investigate a dose-dependence of the observed effects, SH-SY5Y cells were pretreated with increasing concentrations of PC10:0/10:0 and PC20:0/20:0 ranging from 1 to 10 µM with a constant EtOH concentration of 0.2%. PC10:0/10:0 suppressed the cellular H_2_O_2_ release in a dose-dependent manner. H_2_O_2_ release was found to be unaffected by a final PC10:0/10:0 concentration of 1 µM (1 µM PC10:0/10:0 vs. solvent: p = 0.997; 1 µM PC10:0/10:0 vs. 1 µM PC20:0/20:0: p = 0.075), while 3 µM and 10 µM PC10:0/10:0 significantly reduced the cellular H_2_O_2_ release compared to both solvent (3 µM: p = 0.012, 10 µM: p < 1 × 10^–14^) and the identical concentration of PC20:0/20:0 (3 µM: p = 2.74 × 10^–4^, 10 µM: p = 1.84 × 10^–8^) (Fig. 1e). As an additional control the impact of l-α-Glycerophosphorylcholine on the cellular H_2_O_2_ release was measured. l-α-Glycerophosphorylcholine represents the PC head group and the glycerol backbone without attached fatty acids. While the level of H_2_O_2_ released by SH-SY5Y cells was unaffected by 10 µM l-α-Glycerophosphorylcholine compared to solvent (p = 0.891), it was significantly reduced by 10 µM PC10:0/10:0 compared to solvent (p < 1 × 10^–14^), 10 µM l-α-Glycerophosphorylcholine (p < 1 × 10^–14^) and 10 µM PC20:0/20:0 (p < 1 × 10^–14^) (Fig. 1f). These data confirm that the observed effects of PC10:0/10:0 on H_2_O_2_ release are due to the esterified decanoic acid (10:0).

For a more detailed characterisation of the PC10:0/10:0-effect on cellular H_2_O_2_ release, cells were respectively pretreated shortly. As shown in Fig. 1g, 30 min (minutes) short-term incubation of SH-SY5Y cells with 10 µM PC10:0/10:0, 10 µM PC20:0/20:0 or solvent did not affect the level of H_2_O_2_ released by the cells (PC10:0/10:0 vs. solvent: p = 0.262; PC10:0/10:0 vs. PC20:0/20:0: p = 0.231).

### The MCFA decanoic acid (10:0) reduces intracellular ROS levels in neuroblastoma cells and attenuates H_2_O_2_-induced cell death

H_2_O_2_ as small, uncharged molecule crosses cellular membranes by simple diffusion and facilitated diffusion through aquaporin (AQP) 8^5,33^. Accordingly, the suppressed H_2_O_2_ release and the reduced extracellular H_2_O_2_ level of cells treated with PC10:0/10:0 (Fig. 1) might indicate an altered intracellular oxidative stress level. Therefore, the intracellular ROS level was assessed by using the CellROX Green Reagent, which is weakly fluorescent in a reduced state, but exhibits a strong fluorogenic signal upon oxidation by ROS and subsequent binding to deoxyribonucleic acid (DNA).

**Figure 2:**
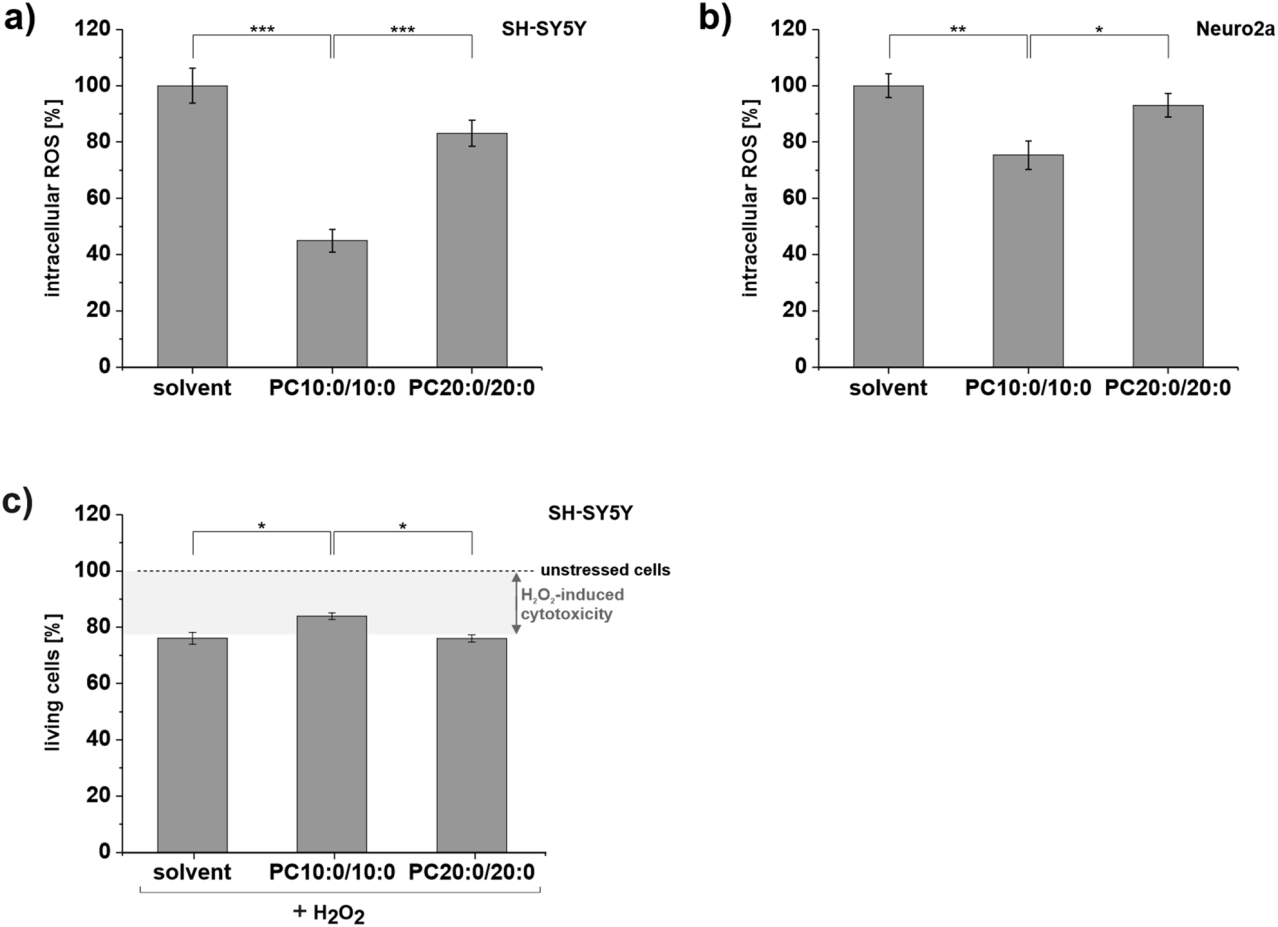
Impact of PC10:0/10:0, PC20:0/20:0 or solvent on the intracellular ROS level and H_2_O_2_-induced cytotoxicity. Cells were incubated with the solvent EtOH (0.2%) (set as 100%), PC10:0/10:0 or PC20:0/20:0 (10 µM) in absence **(a,b)** or presence **(c)** of 500 µM H_2_O_2_ for 18 h before intracellular ROS level and H_2_O_2_-induced changes in cell viability were examined. Intracellular ROS level measured by using CellROX Green in (**a)** SH-SY5Y cells (n = 13) and (**b)** Neuro2a cells (n = 12). (**c)** H_2_O_2_-induced changes in the viability of SH-SY5Y cells determined by labeling dead cells with propidiumiodide (n = 4)**.** Error bars represent SEM. Asterisks show the statistical significance calculated by one-way ANOVA followed by post hoc testing using Tukey's test (* p ≤ 0.05, ** p ≤ 0.01 and *** p ≤ 0.001). Figure was created using Origin Pro 2020b and CorelDRAW Graphics Suite 2020.

In SH-SY5Y cells pretreated with PC10:0/10:0 the intracellular ROS content was significantly reduced compared to solvent (p < 1 × 10^–14^) and PC20:0/20:0 (p = 1.35 × 10^–5^). Again, there was no difference between cells incubated with solvent or PC20:0/20:0 (p = 0.058) (Fig. 2a). In line with this, a significant reduction of the intracellular ROS level was also observed for Neuro2a cells pretreated with PC10:0/10:0 compared to both references (PC10:0/10:0 vs. solvent: p = 1.29 × 10^–3^; PC10:0/10:0 vs. PC20:0/20:0: p = 0.023) (Fig. 2b).

The reduction of intracellular ROS content by PC10:0/10:0 might have neuroprotective effects as excessive ROS levels are associated with the oxidative damage of cellular components and the disruption of cellular integrity^5^. Co-treatment with PC10:0/10:0 indeed protected SH-SY5Y cells from H_2_O_2_-induced cell death. The viability of SH-SY5Y cells exposed to 500 µM H_2_O_2_ for 18 h decreased significantly (p = 1.96 × 10^–5^) compared to control. Co-treatment with PC10:0/10:0 increased viability significantly compared to solvent and PC20:0/20:0 (PC10:0/10:0 + H_2_O_2_ vs. solvent + H_2_O_2_: p = 0.015; PC10:0/10:0 + H_2_O_2_ vs. PC20:0/20:0 + H_2_O_2_: p = 0.014) (Fig. 2c).

### The MCFA decanoic acid (10:0) does not affect cell number and viability

To rule out signal falsifications by general alterations in the number of cells due to treatment procedure, the signal of 4′,6-diamidino-2-phenylindole (DAPI)-labeled nuclei was measured in each well used for the Amplex Red H_2_O_2_ and CellROX Green assays shown above (Fig. 1a–e, Fig. 2a,b). DAPI exhibits a strong fluorescence enhancement upon binding to adenine–thymine-rich regions in the DNA, which correlates with the total number of cells in each well. There were no indications for an effect of PC10:0/10:0 compared to solvent and PC20:0/20:0 on total cell numbers neither for SH-SY5Y nor for Neuro2a cells (Table 1). Similar results were found for the short-term (30 min) pretreatment of SH-SY5Y cells (Supplementary Table S1a). Total cell numbers did also not differ between SH-SY5Y cells incubated with PC10:0/10:0 or l-α-Glycerophosphorylcholine for 18 h (Supplementary Table S1a).

**Table 1:** Impact of PC10:0/10:0, PC20:0/20:0 or solvent on total cell numbers and cell viability.

Total cell number (%)	SH-SY5Y			Neuro2a		
	Solvent	PC10:0/10:0	PC20:0/20:0	Solvent	PC10:0/10:0	PC20:0/20:0
Mean	100.0	103.4	101.6	100.0	96.9	94.7
SEM	2.2	1.8	1.8	1.9	2.2	2.9
Number of independent experiments (n)	53	51	51	24	24	24
p-value solvent vs. PC10:0/10:0	0.424			0.623		
p-value solvent vs. PC20:0/20:0	0.825			0.265		
p-value PC10:0/10:0 vs. PC20:0/20:0	0.789			0.799		

Dead cells (% of total cells)	SH-SY5Y			Neuro2a		
	Solvent	PC10:0/10:0	PC20:0/20:0	Solvent	PC10:0/10:0	PC20:0/20:0
Mean	2.7	2.4	3.3	7.5	8.4	7.3
SD	1.4	1.0	1.2	2.4	4.6	2.4
Number of independent experiments (n)	8	8	7	8	8	7
p-value solvent vs. PC10:0/10:0	0.891			0.853		
p-value solvent vs. PC20:0/20:0	0.644			0.992		
p-value PC10:0/10:0 vs. PC20:0/20:0	0.386			0.800		

Total cell number (%): DAPI signal correlating with total cell number of all wells used for the measurements shown in Figs. 1a–e and 2a,b.

Dead cells (%): the percentage of dead cells after treatment with the solvent EtOH (0.2%), PC10:0/10:0 or PC20:0/20:0 (10 µM) for 18 h was calculated after determination of the total cell number and the number of dead cells utilizing DAPI (1 µg/ml) and Ethidium homodimer-1 (10 µM), respectively.

The statistical significance was calculated by one-way ANOVA followed by post hoc testing using Tukey's test, no significant differences were observed.

*SEM* standard error of the mean, *SD* standard deviation.

The performed DAPI staining indicates the total number of nuclei, but does not allow a differentiation between living and dead cells. Therefore, following 18 h incubation with solvent, 10 µM PC10:0/10:0 or 10 µM PC20:0/20:0 cells were additionally stained with Ethidium homodimer-1, revealing dead cells with a compromised membrane integrity. The number of dead cells was close to equal in all treatments for both cell lines (Table 1).

Thus, the observed effects of PC10:0/10:0 on H_2_O_2_ release and intracellular ROS levels cannot be traced to alterations in the proliferation or viability of the used cells.

### The MCFA decanoic acid (10:0) does not affect βHB levels in neuroblastoma cells

**Figure 3:**
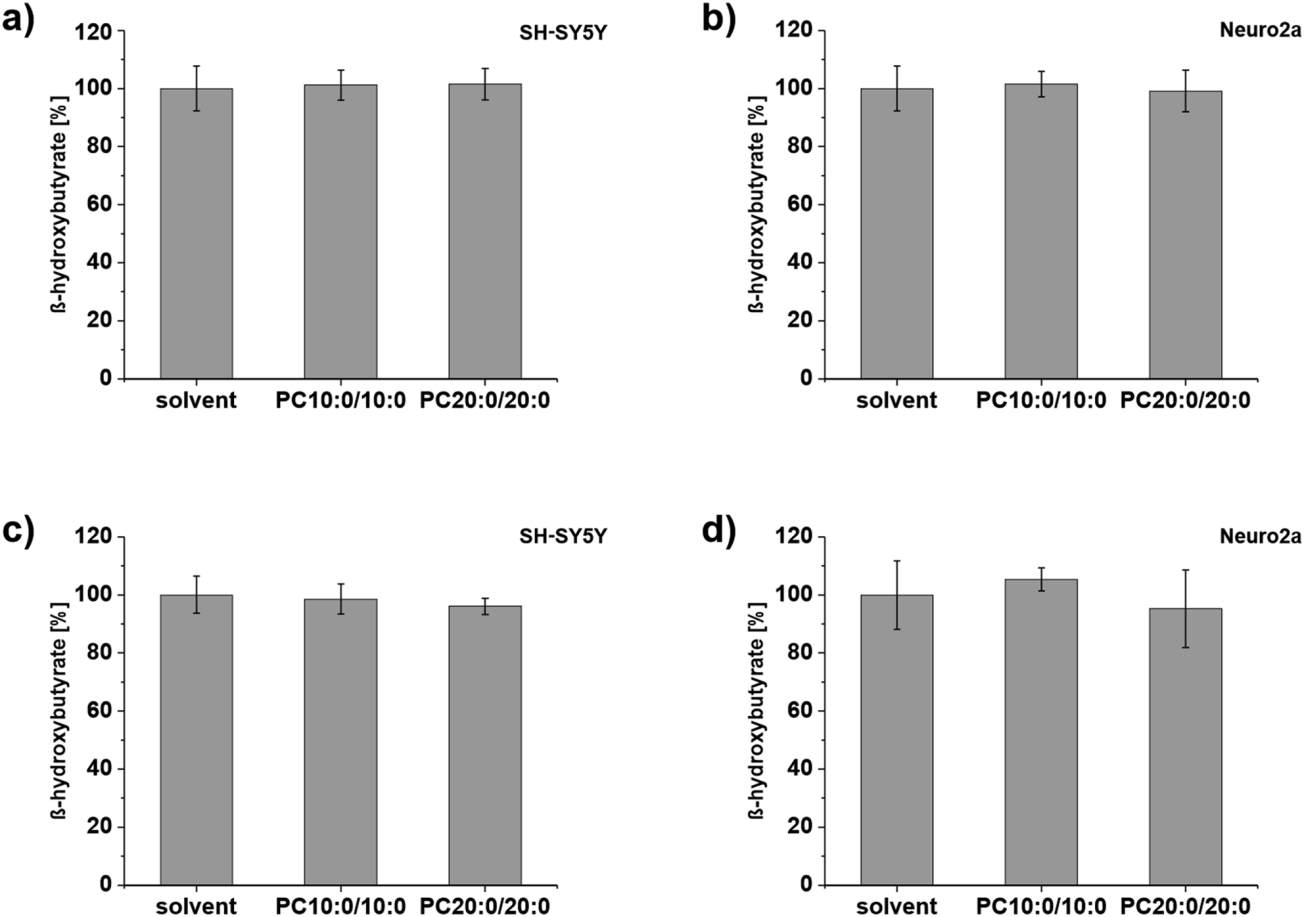
Impact of PC10:0/10:0, PC20:0/20:0 or solvent on the intra- and extracellular βHB level. Cells were incubated with the solvent EtOH (0.2%) (set as 100%), PC10:0/10:0 or PC20:0/20:0 (10 µM) for 18 h before the level of βHB was measured by using the β-Hydroxybutyrate (Ketone Body) Colorimetric Assay Kit. βHB level in the homogenates of (**a)** SH-SY5Y cells (n = 8) and (**b)** Neuro2a cells (n ≥ 5). βHB level in the conditioned medium of (**c)** SH-SY5Y cells (n = 8) and (**d)** Neuro2a cells (n ≥ 5). Error bars represent SD. The statistical significance was calculated by one-way ANOVA followed by post hoc testing using Tukey's test, no significant differences were observed. Figure was created using Origin Pro 2020b and CorelDRAW Graphics Suite 2020.

MCFAs are quickly metabolized into ketone bodies after their dietary intake, in contrast to LCFAs^22^. Ketogenesis occurs primarily in the mitochondria of liver cells, but also in some other cell types including astrocytes^10,34^. It is a process assumed to take place ‘only under very particular physiological circumstances, which are absent in cancer cells’^30^. Accordingly, the use of neuroblastoma cells should allow us to determine the sole effect of the supplemented fatty acids. In order to verify this assumption we measured the level of βHB, the most abundant ketone body in mammals^12^, in the medium and in the homogenates of cells treated with 10 µM PC10:0/10:0, 10 µM PC20:0/20:0 or solvent for 18 h. βHB was detectable within these samples. The intracellular βHB level was found to be unaffected by the different treatments in SH-SY5Y and Neuro2a cells (Fig. 3a,b, Supplementary Table S2a). The βHB content in the cell culture supernatant of the same wells did also not differ between cells treated with PC10:0/10:0, PC20:0/20:0 or solvent for both SH-SY5Y and Neuro2a cells (Fig. 3c,d, Supplementary Table S2a). The total protein content of the homogenates did not vary depending on the different treatments (Supplementary Table S3a). These results indicate the effects of PC10:0/10:0 on H_2_O_2_ release and intracellular oxidative stress levels not to be based on an increased metabolism of decanoic acid (10:0) into ketone bodies, but rather on direct cellular effects of this MCFA.

### The MCFA decanoic acid (10:0) increases catalase activity in SH-SY5Y cells, but not its gene expression

**Figure 4:**
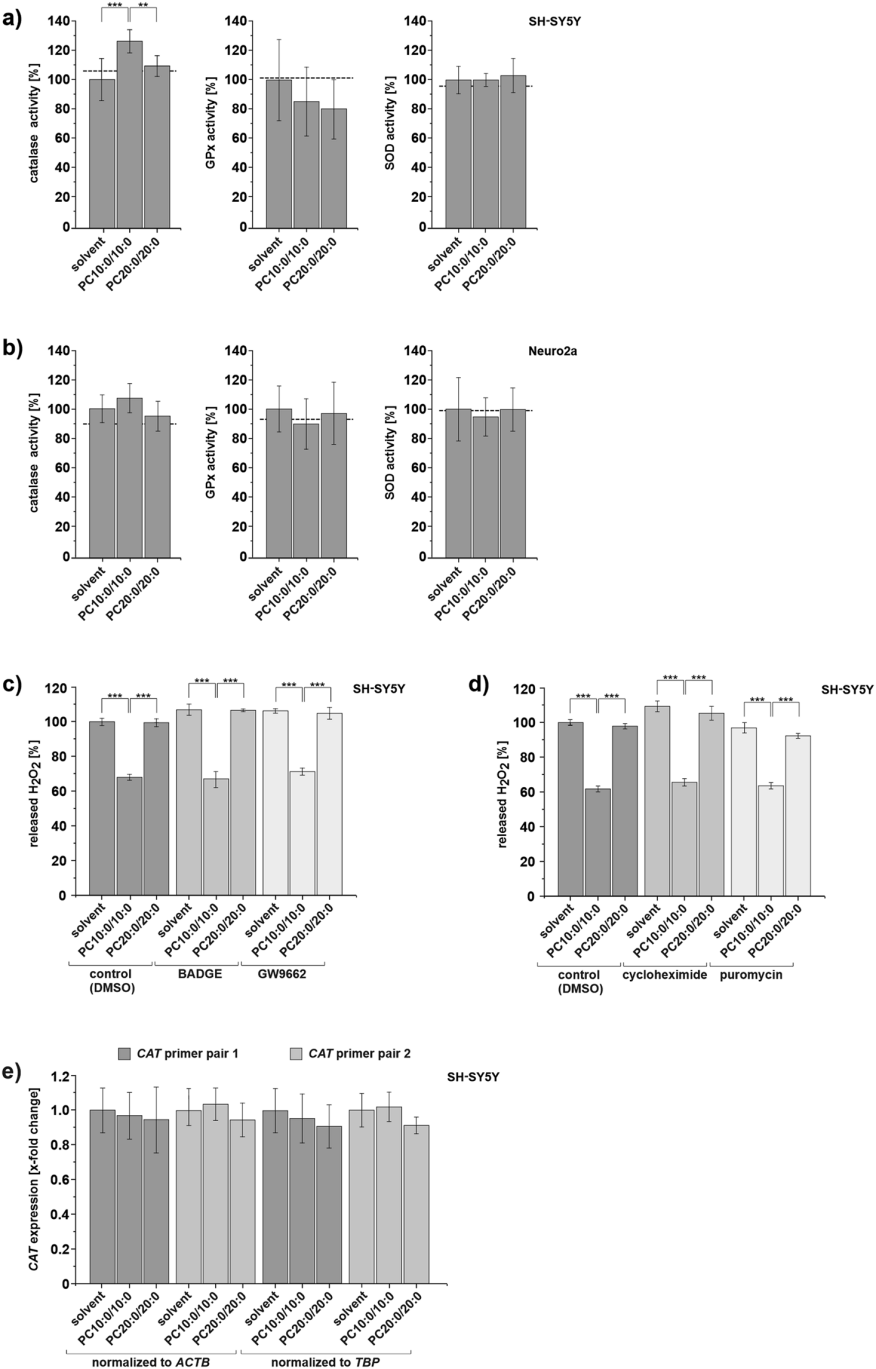
Impact of PC10:0/10:0, PC20:0/20:0 or solvent on the activity of antioxidative enzymes and on catalase gene expression. Cells were incubated with the solvent EtOH (0.2%) (set as 100%), PC10:0/10:0, PC20:0/20:0 (10 µM) for 18 h in absence **(a,b,e)** or presence of PPARγ antagonists **(c)** and inhibitors of protein biosynthesis **(d)**. Catalase, GPx and SOD activity in the homogenates of (**a)** SH-SY5Y cells (catalase: n ≥ 5, GPx: n ≥ 5, SOD: n ≥ 6) and (**b)** Neuro2a cells (catalase: n ≥ 6, GPx: n ≥ 5, SOD: n ≥ 6) determined by using the corresponding enzyme activity assay kits. The activity level of the respective enzyme in untreated cells is indicated by a dotted line. H_2_O_2_ released by SH-SY5Y cells measured by using Amplex Red (5 µM) and HRP (0.01 U/ml) after treatment with phospholipids or solvent along with (**c)** 5 µM BADGE / 5 µM GW9662/ DMSO (n ≥ 4) and (**d)** 20 µM cycloheximide/ 2 µM puromycin/ DMSO (n ≥ 5). (**e)** Catalase gene (*CAT*) expression in SH-SY5Y cells measured by RT-PCR using two different *CAT* primer pairs and two different house keeping genes (*ACTB*: β-actin*, TBP:* TATA-binding protein) for normalization (n = 7). Error bars represent SD **(a,b,e)** or SEM **(c,d).** Asterisks show the statistical significance calculated by one-way ANOVA followed by post hoc testing using Tukey's test (** p ≤ 0.01 and *** p ≤ 0.001). Figure was created using Origin Pro 2020b and CorelDRAW Graphics Suite 2020.

Cellular ROS levels are dependent on the balance between their production and their detoxification by antioxidant enzymes^2–5^. In order to investigate whether ROS-detoxification is affected by decanoic acid (10:0), the activity of catalase, SOD and GPx in SH-SY5Y and Neuro2a cells incubated with PC10:0/10:0, PC20:0/20:0 or solvent for 18 h was measured. The total protein content of the used homogenates was close to equal in all treatments for both cell lines (Supplementary Table S3b). Catalase activity was significantly elevated in SH-SY5Y cells treated with 10 µM PC10:0/10:0 compared to both solvent (p = 6.02 × 10^–5^) and PC20:0/20:0 (p = 0.008). In contrast, SOD- and GPx-activity was unaffected by the different treatments in this cell line (Fig. 4a, Supplementary Table S2b). Thus, the PC10:0/10:0-mediated suppression of H_2_O_2_-release in SH-SY5Y cells might be at least partially based on an upregulation of catalase activity. In Neuro2a cells incubated with PC10:0/10:0 the activity of none of the three analyzed enzymes was significantly altered (Fig. 4b, Supplementary Table S2b).

Hughes et al. reported an upregulation of catalase activity in SH-SY5Y cells after long term-exposure to 250 µM decanoic acid (10:0) as free fatty acid (FFA). This elevation has been ascribed to a peroxisome proliferator-activated receptor (PPAR) γ-dependent increase in catalase gene expression^35^. Accordingly, we investigated whether PC10:0/10:0 similarly affects the enzyme under the conditions chosen in the present study. For this purpose, SH-SY5Y cells were treated with 10 µM PC10:0/10:0, PC20:0/20:0 or solvent in the presence or absence of two different PPARγ antagonists, Bisphenol A diglycidyl ether (BADGE) and GW9662^36,37^. While the total cell numbers were not affected by BADGE, they were slightly decreased after treatment with GW9662 (Supplementary Table S1b). Both PPARγ antagonists did not prevent or attenuate the PC10:0/10:0-mediated reduction in H_2_O_2_ release (Fig. 4c, Supplementary Table S2c). These data indicate that the suppression of H_2_O_2_ release in SH-SY5Y cells treated with PC10:0/10:0 is independent of PPARγ activation. PC10:0/10:0 also significantly reduced the level of H_2_O_2_ released by SH-SY5Y cells irrespective of the presence of cycloheximide and puromycin, two inhibitors of protein biosynthesis^38–41^ (Fig. 4d, Supplementary Table S2c), although both inhibitors significantly affected total cell numbers (Supplementary Table S1b). In line with these results, reverse transcription polymerase chain reaction (RT-PCR), using two different *CAT* primer pairs and two different housekeeping genes for normalization, revealed no differences in *CAT* gene expression in SH-SY5Y cells treated with PC10:0/10:0 compared to solvent and PC20:0/20:0 (Fig. 4e, Supplementary Tables S2d + S4). In conclusion, the increase of catalase activity in SH-SY5Y cells incubated with PC10:0/10:0 seems to be not based on alterations in *CAT* gene expression.

## Discussion

The beneficial effects of dietary MCFAs for cognitively impaired patients have been mainly attributed to their hepatic metabolism resulting in elevated levels of circulating ketone bodies, which positively affect cerebral energy metabolism^8,9,13–23^. In the present study we showed that the MCFA decanoic acid (10:0) might additionally have neuroprotective effects by directly affecting oxidative stress.

Decanoic acid (10:0) administrated in the form of PC10:0/10:0 significantly reduced ROS levels compared to solvent, the PC-backbone l-α-Glycerophosphorylcholine or PC20:0/20:0 containing the LCFA arachidic acid (20:0). This demonstrates the observed effects to be due to the fatty acid chain length and not to the choline head group or the glycerophosphoric acid. Liposomes containing the used PCs have been shown to be taken up by cells in an endocytosis-like manner in nearly equal amounts irrespective of differences in their fatty acid acyl-chain composition. The supplemented PCs are either incorporated into cellular membranes, which might affect membrane structure and fluidity, or hydrolyzed by phospholipases A to lysolipids and FFAs^42^ serving as cellular signal molecules, essential energy sources or precursors of ketones. Under the chosen experimental conditions there seems to be no enhanced metabolism of decanoic acid (10:0) derived from PC10:0/10:0 into ketones. The level of βHB, the most abundant ketone body in mammals^12^, was found to be unaffected by the different treatments. This observation indicates the reduction of oxidative stress in cells treated with PC10:0/10:0 to be independent of ketogenesis. Reduction of ROS in this experimental setup seems to be due to direct effects of decanoic acid (10:0). In line with this, a recent publication by Wang et al*.* provided evidence that a MCT-enriched diet administrated to aged rats has cognition-enhancing properties without altering cerebral ketone levels^24^.

**Figure 5:**
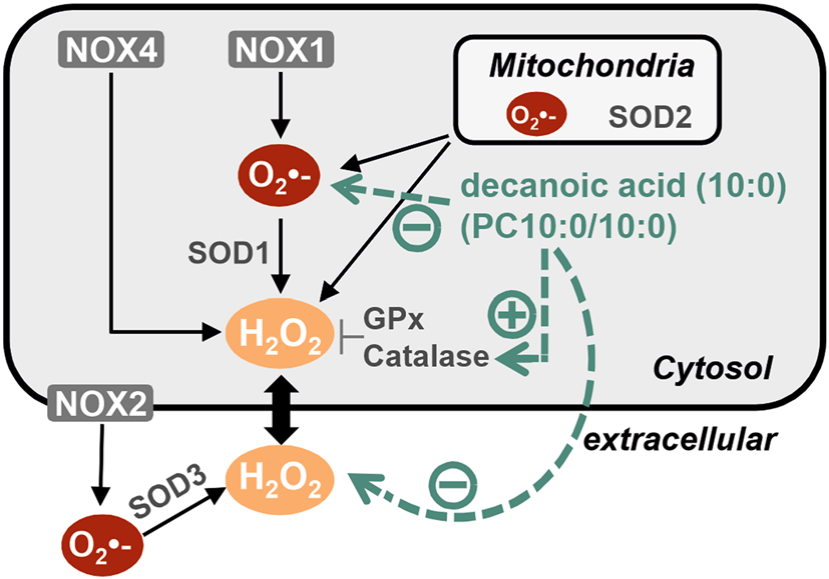
Schematic overview of the cellular generation and detoxification of O_2_^•−^ and H_2_O_2_ including the observed effects of decanoic acid (10:0). The mitochondrial electron transport chain and NOX activity are the major cellular sources of O_2_^•−^, which is rapidly converted to H_2_O_2_ by distinct SODs (mitochondrial SOD2, cytosolic SOD1, extracellular SOD3). In contrast to other NOX isoenzymes generating O_2_^•−^, NOX4 predominatly releases H_2_O_2._ H_2_O_2_ diffuses across cellular membranes and is intracellularly detoxified by antioxidant enzymes such as GPx and catalase. Decanoic acid (10:0) administered in the form of PC10:0/10:0 reduces the intracellullar ROS level with O_2_^•−^ probably representing the major detected ROS species. Additionally, the steady state level of extracellular H_2_O_2_ as well as H_2_O_2_ release is decreased in cells treated with PC10:0/10:0. The latter effect might be at least partially based on an upregulation of catalase activity independent of alterations in *CAT* gene expression. Figure was created using CorelDRAW Graphics Suite 2020.

Accumulation and release of extracellular H_2_O_2_ was measured by using the highly specific and sensitive Amplex Red assay^32,43^, while the intracellular ROS measurements were performed by utilizing CellROX Green. This dye has been reported to represent rather a O_2_^•−^ detector than a general ROS indicator. It does not directly react with H_2_O_2_^44,45^. In conclusion our data suggest that PC10:0/10:0 reduces the levels of both intracellular O_2_^•−^ and extracellular H_2_O_2_ (Fig. 5). O_2_^•−^ is largely generated by the mitochondrial electron transport chain and by the activity of NOX transmembrane proteins. Cytosolic SOD1 and mitochondrial SOD2 rapidly dismutate the intracellular O_2_^•−^ to H_2_O_2,_ which is further detoxified by enzymes such as GPx and catalase (Fig. 5). In line with the data published by Hughes et al*.*^35^, the respective measurement of the enzyme activities revealed a significant elevation of catalase activity in SH-SY5Y cells treated with PC10:0/10:0. Catalase converts H_2_O_2_ into oxygen plus water^46^. The remaining H_2_O_2_ diffuses freely or facilitatedly into the extracellular compartment^5^. Hence, the catalase-stimulating effect of PC10:0/10:0 in SH-SY5Y cells might explain the reduced H_2_O_2_ release in these cells pretreated with PC10:0/10:0. However, PC10:0/10:0 seems to affect also the intracellular O_2_^•−^ concentration and in Neuro2a cells the H_2_O_2_ release without significantly altering catalase activity. This suggests additional PC10:0/10:0-affected mechanisms regulating cellular ROS levels, for example mitochondrial or NOX-dependent O_2_^•−^ generation. Further research is needed to analyse whether also ROS production is affected by PC10:0/10:0 under the chosen conditions and to elucidate the underlying mechanism of action. Decanoic acid (10:0) has been shown to modulate mitochondrial function in neuroblastoma cells. Hughes et al*.* found a marked increase in the mitochondrial citrate synthase, complex I activity and mitochondrial content in SH-SY5Y cells after exposure to 250 µM decanoic acid (10:0), indicating that mitochondrial O_2_^•−^ generation might be altered by this fatty acid. Interestingly, the ketone bodies βHB and AcAc also did not influence the impact of decanoic acid (10:0) on citrate synthase activity^35^. Other authors showed an increase in mitochondrial respiration in mouse skeletal muscle along with a reduced H_2_O_2_ production under a MCFA-rich diet compared to both LCFAs-enriched and control chow. In the same study MCFA (200 µM)-treated C2C12 myotubes displayed increased mitochondrial oxidative capacity and less oxidative stress compared to cells treated with LCFAs^47^. Generally, the biological effects of fatty acids are mediated by their integration into cellular membranes and their function as signal molecules, amongst others. Their incorporation into membrane phospholipids alters the biophysiological properties of cellular membranes such as their fluidity and structure. This has been shown for PUFAs and decanoic acid (10:0) in low micromolar concentrations, likely resulting in a modulating effect on the activity of membrane enzymes, receptors, channels, and transporters^48–50^. Therefore, we speculate that for example the activity of membrane-associated NOX and thus O_2_^•−^ and H_2_O_2_ generation is affected by PC10:0/10:0-treatment. Preferential mediators sensing and transducing FFA signals in neuronal cells are G protein-coupled receptors in the plasma membrane known as Free Fatty Acid Receptors (FFARs), cytosolic Fatty Acid-Binding Proteins (FABPs) and the family of nuclear PPARs including PPARγ, which is directly activated by decanoic acid (10:0)^51,52^. The observation that the reduction of H_2_O_2_ release by PC10:0/10:0 depends on a pretreatment of the cells for more than 30 min, makes the rapid activation of G protein-induced cascades unlikely as mode of action of PC10:0/10:0. In contrast, an impact of PC10:0/10:0-derived decanoic acid (10:0) on transcription factors regulating genes associated with ROS generation and detoxification is conceivable. A similar mode of action has been reported for PUFAs, which stimulate the expression of several antioxidant target genes via an NF-E2-related factor 2 (NRF2)-dependent mechanism^53,54^. Interestingly, Hughes et al*.* showed the decanoic acid (10:0)-mediated increase in catalase activity in SH-SY5Y cells to be completely prevented in the presence of the PPARγ antagonist BADGE (25 µM)^35^. In contrast, we show the PC10:0/10:0-mediated reduction in H_2_O_2_ release to be unaffected by the inhibition of PPARγ or protein biosynthesis in the same cell line. Possible explanations for these divergent results include differences in the concentrations (250 µM decanoic acid vs. 10 µM PC10:0/10:0) and in the administration (FFA vs. fatty acid attached to PC) of the supplemented fatty acids. Although two different PPARγ antagonists were used in the present study revealing similar results, it should be mentioned that also the concentration of BADGE differed (25 µM vs. 5 µM). Further, we found catalase gene expression in SH-SY5Y cells to be unaffected by the treatment with PC10:0/10. Altogether our data indicate that the PC10:0/10:0-mediated reduction of H_2_O_2_ release in SH-SY5Y cells is neither due to an activation of PPARγ nor to alterations in gene expression or the synthesis of new proteins in general. It might be at least partially based on an upregulation of catalase activity, which seems to be independent of alterations in *CAT* gene expression. In addition to the regulation of catalase at the transcriptional and post-transcriptional level, the enzyme has been reported to be regulated by post-translational modifications such as phosphorylation and ubiquitination^55^.

Besides reducing the basal steady-state ROS level, PC10:0/10:0 increased the survival of SH-SY5Y cells exposed to H_2_O_2_-induced oxidative stress. In line with this, Nafar et al*.* reported the pretreatment of primary cortical neurons with either CO or caprylic acid (8:0) (20 µM) or lauric acid (12:0) (50 µM) to protect these neurons from Aβ-induced toxicity. The exposure to Aβ-peptides resulted in an increase of intracellular ROS. The pretreatment of the cells with CO significantly attenuated this^25^. However, the authors did not analyse whether the latter effect is due to MCFAs or rather to the polyphenol content of CO, which is known to have antioxidative and anti-inflammatory properties^56^. The results of our study indicate the ROS-reducing effects of CO described in the study by Nafar et al*.*^25^ to be at least partially mediated by MCFAs and not exclusively by secondary plant ingredients.

In summary, an upregulation of catalase activity in neuronal cells by the MCFA decanoic acid (10:0) has been previously shown. Our study presents the novel finding that this, probably in combination with additional cellular mechanisms, results in the reduction of oxidative stress and in the attenuation of H_2_O_2_-induced cell death. Importantly, these effects seem to be independent of the MCFA metabolization into ketone bodies. Thus, besides secondary plant ingredients, MCFAs might substantially contribute to the neuroprotective and cognition-enhancing effects of CO. Similarly, the beneficial effects of MCTs for cognitively impaired patients might not only be based on increased levels of circulating ketones, but also on the individual antioxidative effects of MCT-derived MCFAs. Latter are able to cross the blood–brain-barrier^57,58^ and might play a role in promoting neuronal health. In addition to various other health effects caused by MCFA intake, also detrimental effects for the myocardium have been reported after excessive MCFA consumption^59–61^. Thus, further studies are needed to assess the potential of MCFAs for pharmacological and/ or nutritional interventions.

## Experimental procedures

### Preparation of lipids

1,2-Didecanoyl-sn-glycero-3-phosphocholine (PC10:0/10:0), 1,2-Di-arachidoyl-sn-glycero-3-phosphocholine (PC20:0/20:0) and l-α-Glycerophosphorylcholine were acquired from Sigma Aldrich (Munich, Germany). Due to the limited solubility of PC20:0/20:0, both phospholipids and L-α-Glycerophosphorylcholine were dissolved in pre-warmed (37 °C) EtOH to a final concentration of 5 mM by vortexing and subsequent sonification for 10 min.

### Cell culture and treatments

Human (SH-SY5Y) and murine (Neuro2a) neuroblastoma cells were maintained in Dulbecco's Modified Eagle Medium (DMEM) containing 10% FCS, 8.25 mM glucose, 1% non-essential amino acid solution, 4 mM l-glutamine, 2 mM sodium pyruvate, penicillin (100 U/ml)/streptomycin (0.1 mg/ml) and 0.05 mg/ml gentamicin in a humidified atmosphere containing 5% CO_2_ at 37 °C. Cells were passed before reaching confluence by detachment with trypsin/EDTA.

Incubation of cells with phospholipids and l-α-Glycerophosphorylcholine was performed as described earlier^62,63^. Briefly, the concentration of FCS was reduced to 0.1% during the treatments in order to reduce the lipid content of the cell culture medium as well as cell proliferation. Cells were kept in FCS-reduced and phenol red–free culture medium (DMEM/ 0.1% FCS) for 6 h prior to the treatments. Then pre-warmed (37 °C) DMEM/ 0.1% FCS was supplemented with phospholipids (10 µM if not stated otherwise), l-α-Glycerophosphorylcholine (10 µM) or the solvent EtOH (0.2%) in glass vials under continuous vortexing and incubated on the cells for 18 h unless specified differently. 5 µM BADGE, 5 µM GW9662, 20 µM cycloheximide, 2 µM puromycin or dimethyl sulfoxide (DMSO) as solvent control were added the incubation medium for the co-treatment of cells with phospholipids and these inhibitors.

### Measurement of extracellular H_2_O_2_ using Amplex Red

Cells were seeded on black 96-well plates (2 × 10^4^ Neuro2a cells/ well, 3 × 10^4^ SH-SY5Y cells/ well) and treated as described above.

For determination of H_2_O_2_ accumulating in the medium during the treatment period, 50 µl conditioned cell culture supernatant of each well were transferred to a new 96-well plate and supplemented with 5 µM Amplex Red (Cayman Chemical, Ann Arbor, USA) and 0.01 U/ml HRP (Thermo Fischer Scientific, Schwerte, Germany). A potential interference between the added agents and the HRP-catalyzed reaction between Amplex Red and H_2_O_2_ was analyzed by repeating this experiment in a cell-free system utilizing freshly prepared DMEM/0.1% FCS + phospholipids (10 µM) or solvent in the presence of supplemented H_2_O_2_ (0.000007%).

For measuring the H_2_O_2_ freshly released by pretreated cells, the supernatant was removed and 50 µl of Amplex Red reaction mixture (5 μM Amplex Red and 0.01 U/ml HRP in phenol red-free DMEM/0.1% FCS) was added to each well.

The fluorescence signal of resorufin was determined at an excitation wavelength of 530 ± 17 nm and an emission wavelength of 590 ± 17 nm for 16 min (120 s (seconds) intervals) at 37 °C under light exclusion in a Safire^2^ Fluorometer (Tecan, Crailsheim, Germany). The increase of fluorescence over time was calculated for each well and used for further data analysis.

### Measurement of intracellular ROS using CellROX Green Reagent

After pretreatment of the cells (2 × 10^4^ Neuro2a cells/ well, 3 × 10^4^ SH-SY5Y cells/ well) as described above, the supernatant was removed and cells were gently washed with prewarmed (37 °C) Ringer's solution (130 mM NaCl, 2.4 mM KCl, 2.5 mM CaCl_2_ × 2 H_2_O, 1.3 mM MgCl_2_ × 6 H_2_O, 10 mM Hepes, 8.25 mM glucose, pH 7.4). Afterwards CellROX Green (Thermo Fischer Scientific, Schwerte, Germany) (1:500/ Ringer's solution) was added to the cells and resulting fluorescence was detected at an excitation wavelength of 485 ± 10 nm and an emission wavelength of 520 ± 10 nm for 15 min (180 s intervals) at 37 °C under light exclusion in a Safire^2^ Fluorometer. The slope of fluorescence over time was calculated for each well and used for further data analysis.

### Measurement of H_2_O_2_-induced cell death

To analyze possible protective effects of PC10:0/10:0 against H_2_O_2_-induced cytotoxicity, SH-SY5Y cells were seeded on 96-well plates (3 × 10^4^ cells/ well) and co-incubated with phospholipids (10 µM) or solvent as described above along with 500 µM H_2_O_2_ for 18 h. Then propidiumiodide, a dye labeling dead cells that have a compromised membrane integrity, was added in a final concentration of 10 µM. After 60 min incubation at 37 °C resulting fluorescence was measured at an excitation wavelength of 510 ± 20 nm and an emission wavelength of 617 ± 20 nm in a Safire^2^ Fluorometer. After the addition of Triton X-100 (0.1%) the signal of 100% dead cells in each well was determined and the percentage of dead and viable cells was calculated for each well.

### Determination of total cell numbers

After determination of extracellular H_2_O_2_ levels, H_2_O_2_ release or intracellular ROS levels in black 96-well plates as described above and removement of the supernatant, cells were fixed with 4% formaldehyde/ phosphate buffered saline (PBS, 2.7 mM KCl, 1.8 mM KH_2_PO_4_, 137 mM NaCl, 10.1 mM Na_2_HPO_4_ × 2 H_2_O) for 15 min and rinsed with PBS. In order to measure the total number of cells in each well DAPI (Cayman Chemical, Ann Arbor, USA) (1 µg/ml in PBS + 0.1% Triton X-100) was added. Resulting fluorescence was detected at an excitation wavelength of 358 ± 15 nm and an emission wavelength of 461 ± 15 nm in a Safire^2^ Fluorometer.

### Determination of cell viability

After the treatment with phospholipids (10 µM) or solvent the percentage of dead cells was assessed by fluorescent microscopy utilizing a DM IL LED microscope (Leica Microsystems, Wetzlar, Germany) and a Retiga R6 camera (Teledyne Photometrics, Birmingham, UK). Cells were stained with Ethidium homodimer-1 (10 µM) (Abcam, Berlin, Germany) and DAPI (1 µg/ml) in Ringer's solution for 10 min in the dark. Ethidium homodimer-1 labels cells with compromised plasma membranes red after binding to DNA. Dead cells showing an overlay of the Ethidium homodimer-1 signal (excitation: 528 nm, emission: 617 nm) and the DAPI signal (excitation: 358 nm, emission: 461 nm) were counted before 0.2% Triton X-100 was added in order to determine the total number of DAPI-stained nuclei. Percentage of dead cells was calculated on the basis of the number of cells showing an overlay of the Ethidium homodimer-1 signal with the DAPI signal and the total number of DAPI-stained nuclei after addition of Triton X-100.

### Measurement of βHB levels

The levels of the ketone body βHB in cell homogenates and in conditioned cell culture medium were determined by using the β-Hydroxybutyrate (Ketone Body) Colorimetric Assay Kit (item no. 700190, Cayman Chemical, Ann Arbor, USA) according to manufacturerʹs instructions with minor modifications^25,64^. Absorbance was monitored in a Safire^2^ Fluorometer. Afterwards the protein content of the cell homogenates was determined as described below.

### Measurement of catalase, SOD and GPx activity

The enzymatic activities of catalase, SOD and GPx were determined by utilizing the Amplex Red Catalase Assay Kit (item no. A22180, Thermo Fischer Scientific, Schwerte, Germany), the Superoxide Dismutase Activity Assay Kit (item no. ab65354, Abcam, Berlin, Germany) and the Glutathione Peroxidase Activity Assay Kit (item no. ab219926, Abcam, Berlin, Germany), respectively. Assays were performed according to manufacturer`s instructions with minor modifications. Resulting absorbance or fluorescence was monitored in a Safire^2^ Fluorometer. Afterwards the protein content of the samples was measured as described below.

### Determination of protein concentration

Protein determination in samples and bovine serum albumin (BSA) standards was carried out using bicinchoninic acid (BCA) according to Smith et al.^65^. Absorbance was measured at a wavelength of 560 nm in a Safire^2^ Fluorometer.

### RT-PCR

Total RNA was extracted by the use of TRIzol Reagent (Thermo Fischer Scientific, Schwerte, Germany). Reverse transcription of RNA was carried out with the RevertAid H Minus First Strand cDNA Synthesis Kit (item no. K1631, Thermo Fischer Scientific, Schwerte, Germany). RT-PCR was performed by utilizing KAPA SYBR FAST (Sigma Aldrich, Munich, Germany) and a CFX Connect Real-Time PCR Detection System (Bio-Rad Laboratories, Feldkirchen, Germany). Results were normalized to β-actin or TATA-binding protein gene expression (*ACTB, TBP*) and changes in catalase (*CAT)* gene expression were calculated by using the 2^−(ΔΔCt)^ method^66^. The following primer sequences were used (Sigma Aldrich, Munich, Germany): *CAT* primer pair 1: 5′-GCTGAGAAGCCTAAGAATGCG-3′ and 5′-GATGAGCGGGTTACACGGAT-3′, *CAT* primer pair 2: 5′- ATTCGATCTCACCAAGGTTTG-3′ and 5′- CTTGGGTCGAAGGCTATCTG-3′, *ACTB:* 5′-CTTCCTGGGCATGGAGTC-3′ and 5′-AGCACTGTGTTGGCGTACAG-3′, *TBP*: 5′-CGGAGAGTTCTGGGATTGT-3′ and 5′-GGTTCGTGGCTCTCTTATC-3′.

### Statistical analyses

Statistical analyses were performed using Origin Pro 2020b software (OriginLab Corporation).

For the measurements of extracellular H_2_O_2_ levels, H_2_O_2_ release, intracellular ROS levels and H_2_O_2_-induced cell death as well as for the subsequent determination of total cell numbers in the corresponding wells, the mean of 3–4 Wells was considered as one independent experiment (n). Accordingly, these data are presented as mean ± standard error of the mean (SEM). All other data are outlined as mean ± standard deviation (SD). Shapiro–Wilk test and Levene's test were used for testing normal distribution and homogeneity of variance, respectively. Statistical significance was determined by one-way analysis of variance (ANOVA) followed by post hoc testing using Tukey's test, significance levels for p-values are as follows: * p ≤ 0.05; ** p ≤ 0.01 and *** p ≤ 0.001.

## Supplementary Information

Supplementary Information.

## Data Availability

The datasets generated during and/or analysed during the current study are available from the corresponding author on reasonable request.
