# Supplementary material for: The medium-chain fatty acid decanoic acid reduces oxidative stress levels in neuroblastoma cells
Source: Sci Rep. 2021 Mar 17;11:6135. doi: 10.1038/s41598-021-85523-9 (PMC7971073; doi:10.1038/s41598-021-85523-9)
Supplement: Supplementary file 1 — Supplementary Information. [file 41598_2021_85523_MOESM1_ESM.pdf]

Supplementary Information

The medium-chain fatty acid decanoic acid reduces oxidative stress levels in neuroblastoma cells

Janine Mett [1, *] and Uli Müller [1]

[1] Biosciences Zoology/Physiology-Neurobiology, ZHMB (Center of Human and Molecular Biology) Faculty NT - Natural Science and Technology, Saarland University, D-66123, Saarbrücken, Germany

***** Correspondence: janine.mett@uni-saarland.de

Supplementary Figure S1

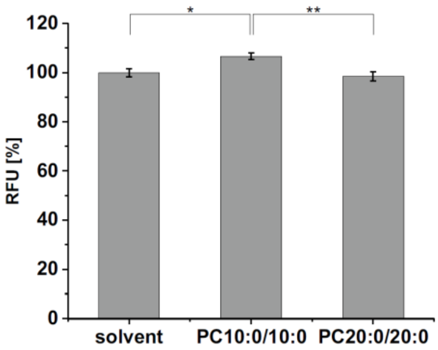

Figure S1: Impact of PC10:0/10:0, PC20:0/20:0 or solvent on the HRP-catalyzed reaction between Amplex Red and H_2O_2 in the cell-free system

DMEM/ 0.1 % FCS containing Amplex Red (5 µM) and HRP (0.01 U/ml) was supplemented with 0.000007 % H_2O_2 in presence of the solvent EtOH (0.2 %) (set as 100 %), PC10:0/10:0 or PC20:0/20:0 (10 µM) (n = 14) before resulting fluorescence was measured (RFU: relative fluorescence units).

Error bars represent SEM. Asterisks show the statistical significance calculated by one-way ANOVA followed by post hoc testing using Tukey's test (* $p \leq 0.05$ and ** $p \leq 0.01$). Figure was created using Origin Pro 2020b and CorelDRAW Graphics Suite 2020.

Supplementary Table S1

Table S1: Total cell numbers of SH-SY5Y cells

Total cell number [%]: DAPI signal correlating with total cell number of the wells used for the measurements shown in Figure 1f + g **(a)** and Figure 4c + d **(b)**. SEM: standard error of the mean. Asterisks show the statistical significance calculated by one-way ANOVA followed by post hoc testing using Tukey's test (* p ≤ 0.05, ** p ≤ 0.01 and *** p ≤ 0.001).

a)

**total cell number [%]**	**SH-SY5Y**				
	**Fig. 1f**		**Fig. 1g**		
	**L-α-Glycero-phosphorylcholine**	**PC10:0/10:0**	**solvent**	**PC10:0/10:0**	**PC20:0/20:0**
mean	100.0	101.7	100.0	99.4	100.0
SEM	2.0	2.0	1.7	1.8	2.1
number of independent experiments (n)	6	6	14	14	14
p-value L-α-Glycerophosphorylcholine vs. PC10:0/10:0	0.567				
p-value solvent vs. PC10:0/10:0			0.969		
p-value solvent vs. PC20:0/20:0			1.000		
p-value PC10:0/10:0 vs. PC20:0/20:0			0.975		

b)

**total cell number [%]**	**SH-SY5Y**					
	**Fig. 4c**			**Fig. 4d**		
	**DMSO**	**BADGE**	**GW9662**	**DMSO**	**cyclo-heximide**	**puromycin**
mean	100.0	98.2	90.0	100.0	76.7	86.8
SEM	2.3	2.6	2.4	2.4	2.4	4.3
number of independent experiments (n)	24	12	18	18	15	18
p-value DMSO vs. BADGE	0.872					
p-value DMSO vs.GW9662	0.009 **					
p-value DMSO vs. cycloheximide				2.65×10^{-5} ***		
p-value DMSO vs.puromycin				0.014 *		

Supplementary Table S2

Table S2: Statistical significances

Additional p-values for the measurements shown in Figure 3a-d **(a)**, Figure 4a + b **(b)**, Figure 4c + d **(c)** and Figure 4e **(d)**. Asterisks show the statistical significance calculated by one-way ANOVA followed by post hoc testing using Tukey's test (* p ≤ 0.05, *** p ≤ 0.001).

a)

**βHB-level**	**SH-SY5Y**		**Neuro2a**	
	**Fig. 3a**	**Fig. 3c**	**Fig. 3b**	**Fig. 3d**
	intracellular βHB	***extracellular βHB***	***intracellular βHB***	***extracellular βHB***
p-value solvent vs. PC10:0/10:0	0.928	0.826	0.926	0.685
p-value solvent vs. PC20:0/20:0	0.878	0.271	0.972	0.738
p-value PC10:0/10:0 vs. PC20:0/20:0	0.992	0.585	0.806	0.251

b)

**enzyme activity**	**SH-SY5Y**		**Neuro2a**		
	**Fig. 4a**		**Fig. 4b**		
	**GPx**	**SOD**	**catalase**	**GPx**	**SOD**
p-value solvent vs. PC10:0/10:0	0.692	1.000	0.420	0.479	0.850
p-value solvent vs. PC20:0/20:0	0.468	0.836	0.710	0.975	1.000
p-value PC10:0/10:0 vs. PC20:0/20:0	0.976	0.843	0.059	0.735	0.835
p-value untreated vs. solvent	0.999	0.906	0.300	0.874	0.999
p-value untreated vs. PC10:0/10:0	0.580	0.901	0.021 *	0.984	0.847
p-value untreated vs. PC20:0/20:0	0.358	0.481	0.803	0.972	0.999

c)

**H_2O_2-release**	**SH-SY5Y**					
	**Fig. 4c**			**Fig. 4d**		
	**DMSO**	**BADGE**	**GW9662**	**DMSO**	**cycloheximide**	**puromycin**
p-value solvent vs. PC10:0/10:0	$< 1 \times 10^{-14}$ ***	3.09×10^{-9} ***	$< 1 \times 10^{-14}$ ***	$< 1 \times 10^{-14}$ ***	$< 1 \times 10^{-14}$ ***	$< 1 \times 10^{-14}$ ***
p-value solvent vs. PC20:0/20:0	1.000	1.000	1.000	0.999	0.962	0.860
p-value PC10:0/10:0 vs. PC20:0/20:0	1×10^{-14} ***	3.76×10^{-9} ***	5.94×10^{-10} ***	$< 1 \times 10^{-14}$ ***	$< 1 \times 10^{-14}$ ***	7.57×10^{-9} ***
p-value PC10:0/10:0 + DMSO vs. PC10:0/10:0 + BADGE	1.000					
p-value PC10:0/10:0 + DMSO vs. PC10:0/10:0 + GW9662	0.982					
p-value PC10:0/10:0 + DMSO vs. PC10:0/10:0 + cycloheximide				0.962		
p-value PC10:0/10:0 + DMSO vs. PC10:0/10:0 + puromycin				1.000		

d)

***CAT* gene expression**	**SH-SY5Y**			
	**Fig. 4e**			
	***CAT* primer pair 1**		***CAT* primer pair 2**	
**reference gene**	***ACTB***	***TBP***	***ACTB***	***TBP***
p-value solvent vs. PC10:0/10:0	0.925	0.799	0.736	0.895
p-value solvent vs. PC20:0/20:0	0.785	0.416	0.534	0.126
p-value PC10:0/10:0 vs. PC20:0/20:0	0.955	0.793	0.185	0.054

Supplementary Table S3

Table S3: Total protein content of cell homogenates

Total protein content [%]: Protein content of cell homogenates used for the measurements shown in Figure 3a-d **(a)** and Figure 4a + b **(b).** SD: standard deviation. The statistical significance was calculated by one-way ANOVA followed by post hoc testing using Tukey's test, no significant differences were observed.

a)

total protein content [%]	SH-SY5Y			Neuro2a		
	Fig. 3a + c			Fig. 3b + d		
	solvent	PC10:0/10:0	PC20:0/20:0	solvent	PC10:0/10:0	PC20:0/20:0
mean	100.0	94.4	96.8	100.0	97.7	100.1
SD	19.2	13.2	12.5	12.3	15.9	7.0
number of independent experiments (n)	8	8	8	5	6	6
p-value solvent vs. PC10:0/10:0	0.745			0.950		
p-value solvent vs. PC20:0/20:0	0.906			1.000		
p-value PC10:0/10:0 vs. PC20:0/20:0	0.948			0.939		

total protein content [%]	SH-SY5Y				Neuro2a			
	Fig. 4a				Fig. 4b			
samples catalase activity	solvent	PC10:0/10:0	PC20:0/20:0	untreated	solvent	PC10:0/10:0	PC20:0/20:0	untreated
mean	100.0	98.1	104.5	93.0	100.0	100.8	90.7	91.8
SD	12.9	9.9	12.4	17.2	10.0	6.3	9.3	12.3
number of independent experiments (n)	8	8	8	5	12	12	12	6
p-value solvent vs. PC10:0/10:0	0.990				0.997			
p-value solvent vs. PC20:0/20:0	0.898				0.085			
p-value PC10:0/10:0 vs. PC20:0/20:0	0.752				0.053			
p-value untreated vs. solvent	0.770				0.302			
p-value untreated vs. PC10:0/10:0	0.896				0.226			
p-value untreated vs. PC20:0/20:0	0.410				0.996			

b)

samples GPx activity	solvent	PC10:0/10:0	PC20:0/20:0	untreated	solvent	PC10:0/10:0	PC20:0/20:0	untreated
mean	100.0	101.3	112.1	92.9	100.0	100.8	90.7	93.7
SD	14.2	9.9	12.0	19.8	10.0	6.3	9.3	12.7
number of independent experiments (n)	5	7	7	6	12	12	12	5
p-value solvent vs. PC10:0/10:0	0.998				0.996			
p-value solvent vs. PC20:0/20:0	0.478				0.083			
p-value PC10:0/10:0 vs. PC20:0/20:0	0.499				0.052			
p-value untreated vs. solvent	0.844				0.584			
p-value untreated vs. PC10:0/10:0	0.715				0.484			
p-value untreated vs. PC20:0/20:0	0.102				0.927			

Supplementary Table S3b continued

samples SOD activity	solvent	PC10:0/10:0	PC20:0/20:0	untreated	solvent	PC10:0/10:0	PC20:0/20:0	untreated
mean	100.0	98.1	104.5	88.5	100.0	100.8	90.7	91.8
SD	12.9	9.9	12.4	18.9	10.0	6.3	9.3	12.3
number of independent experiments (n)	8	8	8	6	12	12	12	6
p-value solvent vs. PC10:0/10:0	0.991				0.997			
p-value solvent vs. PC20:0/20:0	0.910				0.085			
p-value PC10:0/10:0 vs. PC20:0/20:0	0.778				0.053			
p-value untreated vs. solvent	0.403				0.302			
p-value untreated vs. PC10:0/10:0	0.559				0.226			
p-value untreated vs. PC20:0/20:0	0.150				0.996			

Supplementary Table S4

Table S4: RT-PCR cycle threshold (Ct)-values

Ct-values used for the calculation of *CAT* gene expression shown in Figure 4e (n = 7).

CAT: catalase, *ACTB*: β-actin, *TBP*: TATA-binding protein. SD: standard deviation.

Ct-values	SH-SY5Y		
	Figure 4c		
	solvent	PC10:0/10:0	PC20:0/20:0
***CAT* primer pair 1**			
mean	24.28	24.10	24.14
SD	0.16	0.15	0.17
***CAT* primer pair 2**			
mean	25.15	24.87	25.00
SD	0.12	0.21	0.17
ACTB			
mean	18.20	17.98	17.97
SD	0.15	0.18	0.26
TBP			
mean	24.65	24.40	24.37
SD	0.16	0.18	0.23
